# 10 years trends and hospitalization outcomes of non-neonatal tetanus: a large-scale multicenter retrospective study in China

**DOI:** 10.1186/s13054-026-05931-z

**Published:** 2026-03-19

**Authors:** Chunxin Liu, Minli Lei, Jingjing Chen, Miaoting Huang, Yaxin Lu, Yueming Chen, Dengfeng Wu, Xingcai Tan, Yuqiang Liang, Xianwu Zhao, Guowei He, Long Yu, Mingming Huang, Tianrong He, Chi Yang, Gengwei Zhang, Jing Xu, Renfeng Liao, Shaoyun Lu, Shufa He, Huizhong Qiu, Yingjie Chen, Zhong Zhao, Jinrong Liang, Fuxing Xu, Shuyan Liu, Yongxiang He, Yuanjun Lv, Zeqiang Yuan, Huangyang He, Hong Deng, Yingbo Jiang, Weibin Huang, Xianrui Zhuang, Xiaoxin Qiu, Qinhai Liu, Rui Huang, Ke Yang, Yuntao Peng, Yi Ye, Jiajie Ke, Qingli Dou, Wenlong Deng, Haobo Zhang, Xiuqun Kang, Nan Liu, Jun Chen, Wenjian Zhu, Fengqing Song, Yang Liu, Shengnan Xie, Haiyu Gu, Wenming Shao, Licheng Luo, Yaowen Huang, Zenglong Wu, Ping Liu, Maoxiang Liao, Shusen Ye, Xusheng Tu, Jianan Wu, Mufu Wu, Yingquan Chen, Wei Zhang, Haiming Fu, Huarong Ding, Xiao Shao, Anni Lou, Yulin Chen, Zhao Fan, Zengliang Lei, Jianhui Yan, Xian Chen, Na Peng, Wen Lv, Ying Feng, Qing Liang, Mingliang He, Zhilin Huang, Tao Li, Jun Li, Wenfeng Fan, Zhiquan Lai, Yusheng He, Yewen Zhang, Hanzhong Qiu, Yang Yu, Yongguang Huang, Dongjian Liu, Weixin Quan, Huanyao Zhang, Jifeng Li, Liping Wu, Tao Yi, Wensheng Wu, Sheng Xie, Wenhui Huang, Yue Yang, Guangshao Wen, Yan He, Jian Wu, Qunlin Chen, Chuming Liu, Zhixue Hua, Xiaoyan Yang, Keke Wang, Jiasong Hu, Changqing Mao, Jianfei Li, Ping Song, Chunfa Jiang, Weiqin Zhong, Xiaoyue Li, Jialuan Zhang, Bei Hu, Youqing Tang, Zhiming Lian, Li Liu, Wenyue Liu, Qihua Wu, Yaling Chai, Xiaoming Lai, Yong Zheng, Yuanwen Lu, Dianhong Li, Zhipeng Zhou, Qiutian Lin, Guangda Liang, Huayuan Zhu, Feng Zhong, Mingjun Du, Yue Huang, Xingdong Zeng, Huaqing Zhan, Fengxi Wang, Wenzhong Yu, Pengcheng Wu, Ruiquan Wang, Bo Liu, Lintao Yu, Chao Chen, Haidong Wu, Zhenggeng Wu, Lanxiang Wang, Linfeng Zhang, Tengfei Guo, Wenliang Xiao, Guoyan Li, Shaoyi Lin, Fuhua Yan, Yufeng Gao, Yukun Chen, Haixia Wang, Ruisui Chen, Donghua Gu, Weicong Zheng, Cong Liang, Yuetao Zhang, Yanli Wang, Xu Feng, Fuquan Xiao, Xuemin Huang, Guisheng Liao, Yaogen Yuan, Shaofeng Deng, Quanle Liu, Zhenliang Zheng, Yongbiao Zhang

**Affiliations:** 1 Emergency Department, The Third Affiliated Hospital of Sun Yat-Sen University, Guangzhou, China; 2 Data and Artificial Intelligence Center, The Third Affliated Hospital of Sun Yat-Sen University, Guangzhou, Guangdong China; 3 Emergency Department, Central People’s Hospital of Zhanjiang, Zhanjiang, China; 4 Emergency Department, Gaozhou People’s Hospital, Maoming, China; 5 Emergency Department, Guangzhou Eighth People’s Hospital, Guangzhou Medical University, Guangzhou, China; 6 Emergency Department, Maoming People’s Hospital, Maoming, China; 7 Emergency Department, Shantou Central Hospital, Shantou, China; 8 Emergency Department, Qingyuan People’s Hospital, Qingyuan, China; 9 Emergency Department, Zhanjiang Agricultural Reclamation Second Hospital, Zhanjiang, China; 10 Emergency Department, Jiangmen Central Hospital, Jiangmen, China; 11 Emergency Department, Zhongshan City People’s Hospital, Zhongshan, China; 12 Emergency Department, Xinyi People’s Hospital, Maoming, China; 13 Emergency Department, Third People’s Hospital, Shenzhen, China; 14 Emergency Department, The First People’s Hospital of Foshan (Foshan Hospital Affiliated to Southern University of Science and Technology), Foshan, China; 15 Emergency Department, MeiZhou People’s Hospital (MeiZhou City Academy of Medical Sciences), Meizhou, China; 16 Emergency Department, The First People’s Hospital of Zhaoqing, Zhaoqing, China; 17 Emergency Department, Huaiji People′ S Hospital, Zhaoqing, China; 18 Emergency Department, Huizhou Central People’s Hospital, Huizhou, China; 19 Emergency Department, First Affiliated Hospital, Shantou University Medical College, Shantou, China; 20 Emergency Department, Luoding People’s Hospital, Luoding, China; 21 Emergency Department, Xinhui People’s Hospital, Jiangmen, China; 22 Emergency Department, Heyuan People’s Hospital, Heyuan, China; 23 Emergency Department, The Eighth Affiliated Hospital of Southern Medical University (The First People’s Hospital of Shunde), Foshan, China; 24 Emergency Department, Yuebei People’s Hospital, Shaoguan, China; 25 Emergency Department, Kaiping Central Hospital, Jiangmen, China; 26 Emergency Department, Dongguan People’s Hospital, Dongguan, China; 27 Emergency Department, Xinxing People’s Hospital, Yunfu, China; 28 Emergency Department, Lianzhou People’s Hospital, Qingyuan, China; 29 Emergency Department, Southern Theater Command Navy First Hospital, Zhanjiang, China; 30 Emergency Department, Yunfu People’s Hospital, Yunfu, China; 31 Emergency Department, Second Affiliated Hospital of Guangdong Medical University, Zhanjiang, China; 32 Emergency Department, Jieyang People’s Hospital, Jieyang, China; 33 Emergency Department, XingNing People’s Hospital, Meizhou, China; 34 Emergency Department, Zhujiang Hospital of Southern Medical University, Guangzhou, China; 35 Emergency Department, Dianbai People’s Hospital, Maoming, China; 36 Emergency Department, Lufeng People’s Hospital, Shanwei, China; 37 Emergency Department, Nanhai District People’s Hospital, Foshan, China; 38 Emergency Department, Second Affiliated Hospital of Shantou University Medical College, Shantou, China; 39 Emergency Department, The Second Affiliated Hospital of Shenzhen University (Shenzhen Bao’an People’s Hospital), Shenzhen, China; 40 Emergency Department, Dongguan Songshan Lake Central Hospital, Dongguan, China; 41 Emergency Department, Foshan TCM, Foshan, China; 42 Emergency Department, Yuebei Second People’s Hospital, Shaoguan, China; 43 Emergency Department, First Affiliated Hospital of Guangzhou University of Chinese Medicine, Guangzhou, China; 44 Emergency Department, Enping People’s Hospital, Jiangmen, China; 45 Emergency Department, The Affiliated Huizhou Hospital, Guangzhou Medical University, Huizhou, China; 46 Emergency Department, Sun Yat-sen Memorial Hospital, Sun Yat-sen University, Guangzhou, China; 47 Emergency Department, Huadu District People’s Hospital of Guangzhou, Guangzhou, China; 48 Emergency Department, Deqing People’s Hospital, Zhaoqing, China; 49 Emergency Department, Guangzhou First People’s Hospital, Guangzhou, China; 50 Emergency Department, The First Affiliated Hospital of Jinan University, Guangzhou, China; 51 Emergency Department, Taishan People’s Hospital, Jiangmen, China; 52 Emergency Department, Huidong People’s Hospital, Huizhou, China; 53 Emergency Department, Gaoming People’s Hospital, Foshan, China; 54 Emergency Department, Sanshui District People’s Hospital, Foshan, China; 55 Emergency Department, Guangdong Corps Hospital, Chinese People’s Armed Police Force, Guangzhou, China; 56 Emergency Department, Dongguan Hospital of Traditional Chinese Medicine, Dongguan, China; 57 Emergency Department, Fifth Affiliated Hospital of Southern Medical University, Guangzhou, China; 58 Emergency Department, Jiangmen People’s Hospital, Jiangmen, China; 59 Emergency Department, Yangdong People’s Hospital, Yangjiang, China; 60 Emergency Department, Dongguan Tungwah Hospital, Dongguan, China; 61 Emergency Department, Longchuan People’s Hospital, Heyuan, China; 62 Emergency Department, Chaozhou People’s Hospital, Chaozhou, China; 63 Emergency Department, Guangdong Agribusiness Central Hospital, Zhanjiang, China; 64 Emergency Department, Southern Medical University Nanfang Hospital, Guangzhou, China; 65 Emergency Department, Panyu District Central Hospital, Guangzhou, China; 66 Emergency Department, Peking University Shenzhen Hospital, Shenzhen, China; 67 Emergency Department, Wengyuan People’s Hospital, Shaoguan, China; 68 Emergency Department, Dongguan Binhai Bay Central Hospital, Dongguan, China; 69 Emergency Department, Shunde Traditional Chinese Medicine Hospital, Foshan, China; 70 Emergency Department, General Hospital of Southern Theater Command, Guangzhou, China; 71 Emergency Department, Shenzhen People’s Hospital, Shenzhen, China; 72 Emergency Department, The Fifth Affiliated Hospital, Sun Yat-sen University, Zhuhai, China; 73 Emergency Department, First Affiliated Hospital of Guangzhou Medical University, Guangzhou, China; 74 Emergency Department, Jiangmen Wuyi Hospital of TCM (Affiliated Jiangmen TCM Hospital of Ji’nan University), Jiangmen, China; 75 Emergency Department, Nanxiong People’s Hospital, Shaoguan, China; 76 Emergency Department, Kanghua Hospital of Dongguan, Dongguan, China; 77 Emergency Department, ZhuhaiHospital of Integrated Traditional Chinese and Western Medicine, Zhuhai, China; 78 Emergency Department, Fogang People’s Hospital, Qingyuan, China; 79 Emergency Department, Xiaolan People’s Hospital of ZhongShan (The Fifth People’s Hospital of ZhongShan), Zhongshan, China; 80 Emergency Department, Sixth People’s Hospital of Huizhou, Huizhou, China; 81 Emergency Department, Fengkai People’s Hospital, Zhaoqing, China; 82 Emergency Department, Shaoguan First People’s Hospital, Shaoguan, China; 83 Emergency Department, Qingyuan Traditional Chinese Medicine Hospital, Qingyuan, China; 84 Emergency Department, Foshan Second People’s Hospital, Foshan, China; 85 Emergency Department, Luoding Hospital of Traditional Chinese Medicine, Luoding, China; 86 Emergency Department, Suixi County Hospital, Zhanjiang, China; 87 Emergency Department, The Fourth Affiliated Hospital of Guangzhou Medical University (Zengcheng District People’s Hospital), Guangzhou, China; 88 Emergency Department, Second People’s Hospital of Shantou, Shantou, China; 89 Emergency Department, Yangjiang People’s Hospital, Yangjiang, China; 90 Emergency Department, Longgang Central Hospital, Shenzhen, China; 91 Emergency Department, Shanwei People’s Hospital, Shanwei, China; 92 Emergency Department, Boluo County People’s Hospital, Huizhou, China; 93 Emergency Department, Rongcheng City Central Hospital, Jieyang, China; 94 Emergency Department, Longhu People’s Hospital, Shantou, China; 95 Emergency Department, Dabu Counrty People’s Hospital, Meizhou, China; 96 Emergency Department, Zhuhai People’s Hospital, Zhuhai, China; 97 Emergency Department, Pengpai Memorial Hospital, Shanwei, China; 98 Emergency Department, Shanwei Yihui Fund Hospital (The Second People’s Hospital of Shanwei), Shanwei, China; 99 Emergency Department, Qingcheng District People’s Hospital, Qingyuan, China; 100 Emergency Department, Yangshan County People’s Hospital, Qingyuan, China; 101 Emergency Department, Chaozhou Central Hospital, Chaozhou, China; 102 Emergency Department, First Affiliated Hospital of SunYat-sen University, Guangzhou, China; 103 Emergency Department, Second Affiliated Hospital of Guangzhou Medical University, Guangzhou, China; 104 Emergency Department, SunYat-sen University Affiliated, Third Hospital Yuedong Hospital, Meizhou, China; 105 Emergency Department, The Second People’s Hospital of Zhaoqing, Zhaoqing, China; 106 Emergency Department, Shenzhen Children′s Hospital, Shenzhen, China; 107 Emergency Department, Houjie Hospital of Dongguan, Dongguan, China; 108 Emergency Department, Maonan District People’s Hospital, Maoming, China; 109 Emergency Department, Fifth Affiliated Hospital of Zunyi Medical College, Zhuhai, China; 110 Emergency Department, Yangshan County Traditional Chinese Medicine Hospital, Qingyuan, China; 111 Emergency Department, Guangdong Provincial People’s Hospital (Guangdong Academy of Medical Sciences), Southern Medical University, Guangzhou, China; 112 Emergency Department, Guangdong Second People’s Hospital, Guangzhou, China; 113 Emergency Department, Guangzhou Hospital of Integrated Chinese and Western Medicine, Guangzhou, China; 114 Emergency Department, Chenghai District People’s Hospital, Shantou, China; 115 Emergency Department, Jiaoling People’s Hospital, Meizhou, China; 116 Emergency Department, Guangning County People’s Hospital, Zhaoqing, China; 117 Emergency Department, Shenzhen Longhua People’s Hospital, Shenzhen, China; 118 Emergency Department, Lianping County People’s Hospital, Heyuan, China; 119 Emergency Department, Qingxin District People’s Hospital, Qingyuan, China; 120 Emergency Department, Yingde City Hospital of Traditional Chinese Medicine, Qingyuan, China; 121 Emergency Department, Guangdong Second Hospital of Traditional Chinese Medicine, Guangzhou, China; 122 Emergency Department, Guangzhou Red Cross Hospital, Guangzhou, China; 123 Emergency Department, Traditional Chinese Medicine Hospital of PuningCity, Jieyang, China; 124 Emergency Department, Yangjiang Hospital of Traditional Chinese Medicine, Yangjiang, China; 125 Emergency Department, Fengshun County People’s Hospital, Meizhou, China; 126 Emergency Department, Qishi Hospital of Dongguan, Dongguan, China; 127 Emergency Department, The Sixth Affiliated Hospital of Jinan University, Dongguan, China; 128 Emergency Department, Foshan Fifth People’s Hospital, Foshan, China; 129 Emergency Department, Rulin Bay Integrated Traditional Chinese and Western Medicine Hospital of Nanhai District, Foshan, China; 130 Emergency Department, The Third People’s Hospital of Shunde, Foshan, China; 131 Emergency Department, Leliu Hospital of Shunde, Foshan, China; 132 Emergency Department, Yunan County People’s Hospital, Yunfu, China; 133 Emergency Departmentt, Zhongshan Torch Development Zone People’s Hospital, Zhongshan, China; 134 Emergency Department, First Affiliated Hospital of Guangdong Pharmaceutical University, Guangzhou, China; 135 Emergency Department, Third Affiliated Hospital of Guangzhou Medical University, Guangzhou, China; 136 Emergency Department, Third Affiliated Hospital of Southern Medical University, Guangzhou, China; 137 Emergency Department, Baiyun Hospital of The First Affiliated Hospital of Guangzhou University of Chinese Medicine, Guangzhou, China; 138 Emergency Department, Eighth Affiliated Hospital of Sun Yat-sen Uni versity, Shenzhen, China; 139 Emergency Department, Southern University of Science and Technology Hospital, Shenzhen, China; 140 Emergency Department, Shenzhen Luohu People’s Hospital, Shenzhen, China; 141 Emergency Department, Zhangmutou Hospital of Dongguan, Dongguan, China; 142 Emergency Department, Fenggang Hospital of Dongguan, Dongguan, China; 143 Emergency Department, Shijie Hospital of Dongguan, Dongguan, China; 144 Emergency Department, Nanhai District Hospital of TCM, Foshan, China; 145 Emergency Department, Longjiang Hospital of Shunde, Foshan, China; 146 Emergency Department, Zhongshan Guzhen People’s Hospital, Zhongshan, China; 147 Emergency Department, Guangzhou twelfth People’s Hospital, Guangzhou, China; 148 Emergency Department, Sixth Affiliated Hospital of Sun Yat-sen University, Guangzhou, China; 149 Emergency Department, Huiya Hospital of the First Affiliated Hospital of Sun Yat-sen University, Huizhou, China; 150 Emergency Department, Dinghu District People’s Hospital, Zhaoqing, China; 151 Emergency Department, Zhaoqing Gaoyao District Traditional Chinese Medicine Hospital, Zhaoqing, China; 152 Emergency Department, Guangning County Hospital of Traditional Chinese Medicine, Zhaoqing, China; 153 Emergency Department, The Second People’s Hospital of Fengkai, Zhaoqing, China; 154 Emergency Department, Fengkai Hospital of Traditional Chinese Medicine, Zhaoqing, China; 155 Emergency Department, University of Hong Kong-Shenzhen Hospital, Shenzhen, China; 156 Emergency Department, Longgang People’s Hospital, Shenzhen, China; 157 Emergency Department, Wengyuan County Hospital of Traditional Chinese Medicine, Heyuan, China; 158 Emergency Department, Xinfeng People’s Hospital, Shaoguan, China; 159 Emergency Department, Dongguan Eighth People’s Hospital, Dongguan, China; 160 Emergency Department, Gaobu Hospital of Dongguan, Dongguan, China; 161 Emergency Department, Qiaotou Hospital of Dongguan, Dongguan, China; 162 Emergency Department, The Second Affiliated Hospital of Guangzhou University of Chinese Medicine, Zhuhai, China; 163 Emergency Department, Dongyuan County People’s Hospital, Heyuan, China

**Keywords:** Tetanus, Epidemiology, Retrospective study, Hospitalization, Critical care

## Abstract

**Objectives:**

Non-neonatal tetanus continues to pose a public health challenge in low- and middle-income regions. This study aimed to describe the decade-long profile of non-neonatal tetanus in Guangdong, China, from 2011 to 2021—a period marked by rapid economic development and healthcare system strengthening.

**Design:**

Multicenter, retrospective study.

**Setting:**

The study was conducted across 159 hospitals in Guangdong, China.

**Patients:**

We included patients with a clinical diagnosis of tetanus admitted between 2011 and 2021. A total of 2,567 cases (median age 53 years; 62.1% male) were analyzed.

**Interventions:**

None.

**Measurements and main results:**

Data on demographics, vaccination status, clinical severity (Ablett classification), etiology, seasonal trends, critical care utilization, outcomes, and costs were collected. Farmers constituted the largest occupational group (43.7%), and only 2.2% had documented tetanus vaccination. Hospitalizations fluctuated but showed an overall increase, with a rising proportion of severe cases (Ablett grade III/IV). A seasonal peak was observed in August–September. The most common etiology was post-traumatic injury (43.4%). Critical care was frequently required: 25.1% of patients were admitted to the ICU, and 23.8% received mechanical ventilation. Multivariable analysis identified higher Ablett grade, injection drug use, unknown etiology, and comorbidities as independent risk factors for mortality; ICU admission was protective. Case fatality declined significantly from 11.8% in 2012 to 3.5% in 2021, while median hospitalization costs rose substantially.

**Conclusions:**

This large-scale study reveals a substantial burden of severe non-neonatal tetanus among under-vaccinated adults, persisting despite the region’s economic transition. Case fatality decreased over the study period, likely reflecting improvements in both the accessibility and quality of critical care, albeit with increasing healthcare costs. The association between injection drug use or unknown etiology and mortality warrants heightened clinical vigilance.

**Supplementary Information:**

The online version contains supplementary material available at 10.1186/s13054-026-05931-z.

## Key points

- **Question**: How has the clinical profile of non-neonatal tetanus evolved in a region undergoing rapid economic development and healthcare system transition?
- **Findings**: In a large, multicenter retrospective study of 2,567 patients in Guangdong, China (2011-2021), a substantial burden of severe disease was observed among under-vaccinated adults (only 2.2% with documented vaccination), despite major economic development. Case fatality significantly declined from 11.8% to 3.5%, coinciding with a high rate of critical care utilization (25.1% ICU admission), but median hospitalization costs rose substantially. High mortality was linked to disease severity (Ablett grade), injection drug use, and unknown etiology.
- **Meaning**: Economic development alone does not eliminate tetanus risk among under-vaccinated adults. The observed decline in case fatality, coinciding with high ICU utilization, highlights the life-saving role of enhanced critical care access. Future efforts need to pair adult vaccination campaigns with sustained critical care investment to both reduce incidence and improve survival, especially in identified high-risk groups.

**Supplementary Information:**

The online version contains supplementary material available at 10.1186/s13054-026-05931-z.

## Introduction

Tetanus, a life-threatening neurological disease caused by the potent neurotoxin of *Clostridium tetani*, remains a significant yet preventable public health challenge. While global elimination campaigns have drastically reduced maternal and neonatal tetanus, the burden of non-neonatal tetanus persists, particularly in low- and middle-income regions. In 2023, 21 830 cases of tetanus were reported to the World Health Organization (WHO), with 80% occurring in non-neonates [1]. However, this figure is believed to be a substantial underestimate due to systemic surveillance weaknesses in many high-burden countries where tetanus is not a notifiable disease; disease modelling suggests the true global mortality may be as high as 50,000 deaths annually [2]. Tetanus fatality rates are significantly higher in low- and middle-income countries compared to high-income ones, due to a lack of intensive care access and facilities [3]. Therefore, the changing profile of tetanus must be interpreted against the backdrop of socioeconomic development and health system strengthening.

While there have been numerous reports on the characteristics and management of non-neonatal tetanus patients, these studies have generally been limited by small sample sizes [4–8]. Furthermore, although research exists from countries and regions at different economic levels, few studies have tracked the evolution of tetanus management within a single region undergoing rapid economic transformation.

China’s Guangdong Province presents an ideal case study for investigating this dynamic. As the nation’s most populous and economically vibrant region, Guangdong underwent a pivotal transition from a middle-income to a high-income economy during the 2011–2021 study period [9, 10]. This remarkable growth was paralleled by massive investment in public health and intensive care facilities [11]. This transformative decade offers a unique natural experiment to examine how the epidemiology and clinical management of a vaccine-preventable disease like tetanus evolve in tandem with economic advancement and healthcare infrastructure enhancement.

To address this evidence gap, we conducted a large-scale, multicenter, retrospective study, analyzing data from more than 2,000 clinically diagnosed tetanus patients admitted to 159 general hospitals across Guangdong Province between 2011 and 2021. To our knowledge, this represents the largest clinical dataset of its kind. Our study aims to:1) describe the clinical characteristics of non-neonatal tetanus in a highly populated, rapidly developing region; 2) analyze temporal trends in hospitalized tetanus cases, case fatality, healthcare resource utilization, and associated costs; 3) identify factors independently associated with treatment outcomes in a real-world setting.

## Method and methods

### Study design

This was a multicenter, retrospective, observational study of patients hospitalized for tetanus in Guangdong Province, China from January 2011 to July 2021. Guangdong Province comprises 21 prefecture-level cities. There are approximately 632 secondary and tertiary hospitals in the province. After excluding all specialized hospitals (e.g., oncology and children’s hospitals), we identified 485 eligible general hospitals as our target for invitation. Among these, 291 hospitals (60.0%) responded with agreement to participate. Ultimately, 162 hospitals (99 tertiary and 63 secondary hospitals) provided data for medical record abstraction. Following the exclusion of duplicate records and cases not meeting the inclusion criteria, data from 159 hospitals were included in the final analysis.

### Data collection

Access to inpatient medical records for patients diagnosed with tetanus was obtained from the electronic Hospital Information System (HIS) of each participating hospital. Trained research coordinators used a standardized data abstraction form to extract the required variables from the local HIS. Data was encoded using unique patient codes and entered into a database.

The definition of tetanus patients was taken from the terminology of the International Classification of Diseases, 10th Revision (ICD-10), based on the final clinical diagnosis at hospital discharge.

The demographic characteristics of the tetanus patients, such as gender, age, occupation, tetanus vaccination history, and comorbidities, were recorded. In terms of clinical presentation, presumed etiology of tetanus, site of injury or infection, interval from exposure to onset, interval from onset to initial visit, presenting symptoms, clinical forms of tetanus, and grading of disease severity, with/without autonomic nervous disorders, et al. were recorded. In terms of clinical management, medications (such as anti-tetanus immunoglobulin, tetanus vaccination, antibiotics, sedatives, muscle relaxants) and whether mechanical ventilation and tracheotomy involved in patient care were noted. Data on the clinical course was recorded in terms of duration of hospital and intensive care unit (ICU) stay, complications encountered. Lastly, hospitalization expenses and outcome of admission were also included. Hospitalization expenses were extracted from the financial settlement module of the HIS, specifically the "total hospitalization expenses" field from the final inpatient expense summary.

The entire dataset from all sites was reviewed independently by two central researchers (LML, HMT). Any inconsistencies or discrepancies identified during this independent review were resolved through mutual discussion and, when necessary, by re-consulting the original electronic medical record at the respective hospital. Duplicate records (retaining only the final treating hospital’s record) and cases with unresolvable data errors were excluded from the final analysis.

### Operational definitions

To ensure clarity and reproducibility, the following key terms were defined according to standardized criteria:

- 1. Clinical Forms of Tetanus
- Localized tetanus: Muscle spasms confined to the site of injury; Cephalic tetanus: Occurs after head trauma, manifesting as facial and pharyngeal muscle spasms; Generalized tetanus: Generalized muscle rigidity and spasms, the most common form.
- 2. Disease Severity (Ablett Classification)
- Grade I (Mild): Mild to moderate trismus, no respiratory compromise, no or mild dysphagia; Grade II (Moderate): Moderate trismus, transient convulsive seizures, dyspnea (respiratory rate > 30/min), mild dysphagia; Grade III (Severe): Severe trismus, generalized spasms, severe dyspnea (respiratory rate > 40/min), severe dysphagia, tachycardia > 120/min; Grade IV (Very Severe): Grade III criteria plus severe autonomic instability (e.g., alternating hypertension/tachycardia and hypotension/bradycardia).
- 3. Etiology Classification
- Post-traumatic: Following acute trauma (e.g., puncture wounds, fractures, burns); Non-traumatic infectious: Secondary to chronic infections or lesions (e.g., chronic ulcers, otitis media, postoperative infections); Injection drug use-associated: Related to contamination of injected drugs or equipment; Unknown: No identifiable history of trauma or infectious focus.
- 4. Comorbidities were defined as pre-existing conditions not directly caused by tetanus. The full list of recorded comorbidities is provided in Table 1.

**Table 1:** Demographic characteristics of tetanus patients

Variable	Number (n)	Percentage (%)
**Gender**		
Female	973	37.9%
Male	1594	62.1%
**Age, year**		
14 ~ 17	39	1.5%
18 ~ 45	539	21.0%
46 ~ 65	1143	44.5%
66 ~ 79	560	21.8%
> 79	286	11.2%
**Occupation**		
Student	27	1.1%
Labor workers	341	13.3%
Farmer	1123	43.7%
Other professions	483	18.8%
The unemployed	516	20.1%
Retiree	77	3.0%
**Tetanus Vaccination history**		
Yes	55	2.2%
None	298	11.6%
Unknown	2214	86.2%
**Comorbidities**		
None	1690	65.8%
Malignancy	43	1.7%
Hypertension or other cardiovascular disease	310	12.1%
Pulmonary disease	60	2.3%
Diabetes mellitus or other endocrine disease	96	3.7%
Stroke or other neurologic disease	78	3.0%
Renal disease	10	0.4%
Liver and Biliary Diseases	69	2.7%
HIV and other immunodeficiency disorders	8	0.3%
Others	203	7.9%

HIV = Human Immunodeficiency Virus

Values are number or percentage. Age is also presented as median (interquartile range, IQR)

### Statistical analysis

The statistical analysis was performed using R version 3.6.2 software (Institute for Statistics and Mathematics, Vienna, Austria; www.r-project.org). Quantitative variables are expressed as mean ± standard deviation (SD) or the median and interquartile range (IQR), and qualitative variables are presented as numbers (percentage).

Given the low proportion of missing data (< 5%), missing values were addressed using single imputation prior to further analysis: continuous variables were imputed with the mean, and categorical variables were imputed with the mode.

For multivariable analysis, variables with a p-value < 0.05 in univariate analysis were considered for inclusion. Age and sex were forced into the model regardless of univariate significance due to their clinical relevance. Multicollinearity was assessed using variance inflation factors (VIF), with all VIF values < 5 indicating no serious concerns.

To account for potential clustering effects at the hospital level, generalized estimating equations (GEE) analysis was used to assess the risk factors associated with fatality. Both univariate and multivariable GEE models were performed, with hospital ID specified as the clustering variable. A *P* value < 0.05 was considered statistically significant.

## Results

### Temporal and seasonal patterns of tetanus cases

A total of 159 general hospitals participated in this study. The geographic distribution of these hospitals across all 21 prefecture-level cities of Guangdong Province is shown in Supplementary Fig. 1. The study included patients aged over 14 years, as younger individuals are typically admitted to specialized pediatric hospitals and were therefore excluded. From January 2011 to July 2021, 2,567 tetanus cases were identified.

**Fig. 1:**
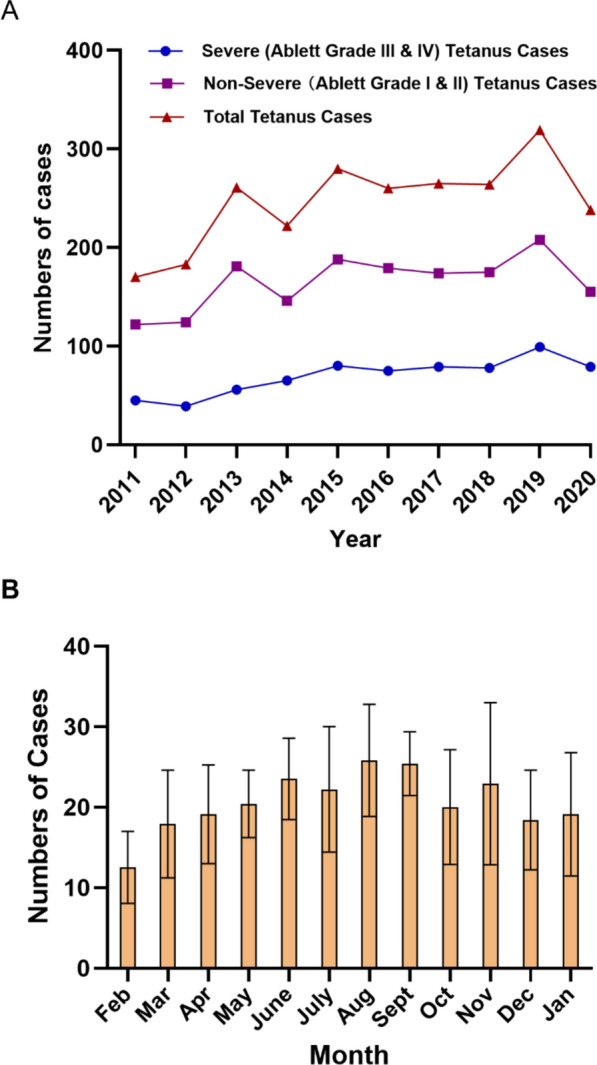
Temporal and Seasonal Distribution of Tetanus Cases (2011–2020). (**A**) Annual trend in the number of tetanus cases. (**B**) Monthly distribution of tetanus case admissions. Each analysis was repeated three times. Data were presented as the mean ± SD. Data from 2021 were excluded from this figure owing to the inclusion of only seven months of data

To ensure standardized comparison of annual trends, the analysis was restricted to complete years from 2011 to 2020. During this period, the annual case count fluctuated with an overall increasing trend, ranging from a low of 170 in 2011 to a peak of 319 in 2019. A more consistent and pronounced increase was observed in both the number and proportion of severe (Grade III + IV) tetanus cases, with the proportion being lowest in 2012 (21.3%) and highest in 2020 (33.2%) (Fig. 1).

A clear seasonal pattern was evident, with the lowest number of cases occurring in February. Case counts rose steadily thereafter, peaking in the late summer months of August and September. (Fig. 1).

### Demographic characteristics of tetanus cases

Among the 2,567 tetanus cases, the majority (62.1%) were male, resulting in a male-to-female ratio of 1.7:1. The median age of the patients was 53 years (IQR, 42–64). The largest proportion of patients (44.5%) belonged to the 46–65 age group, while the elderly (aged > 65 years) representing one-third of the cohort.

An analysis of occupational distribution identified farmers as the largest subgroup, comprising 43.7% of the cohort. Notably, only 2.2% of patients had a confirmed tetanus vaccination history; the remaining 97.8% had either no or an unknown immunization status. Regarding comorbidities, most patients (65.8%) had no documented pre-existing conditions. The detailed demographic data are presented in Table 1.

### Clinical presentation

Table 2 summarizes the clinical characteristics of the 2,567 hospitalized tetanus patients upon presentation. The most common etiology was post-traumatic (1,115 patients, 43.4%), followed by non-traumatic infections (912, 35.5%). A smaller proportion of cases were attributed to injection drug use (0.5%, n = 11), while the etiology remained unknown in 20.6% of cases. The lower extremities were the most common site of injury or infection (48.7%). The median interval from exposure to symptom onset (incubation period) was 10 days (IQR: 6–20), while the median time from symptom onset to the initial hospital visit was 3 days (IQR: 2–7). Although the reported incubation period for tetanus typically ranges from 3 to 21 days [12], our data revealed that 4.7% of patients had an incubation period of ≤ 2 days, and 11.8% had an incubation period of ≥ 30 days.

**Table 2:** Clinical presentation of tetanus patients

Variable	Number(n)	Percentage (%)
**Etiology of Tetanus**		
Post-traumatic	1115	43.4%
Non-traumatic infection	912	35.5%
Injection drug use	11	0.5%
Unknown etiology	529	20.6%
**Site of Injury or Infection**		
Head and face	194	(7.56%)
Trunk	52	(2.03%)
Upper extremities	558	(21.7%)
Lower extremities	1249	(48.7%)
Perineal area	2	(0.08%)
Others	80	(3.12%)
Unknown	432	(16.8%)
**Interval from exposure to onset, in days (median, IQR)**	10 (7—20)	
**Interval from onset to initial visit, in days (median, IQR)**	3 (2—7)	
Presenting symptom		
Trismus	2336	91%
Dysphagia	1622	63.2%
Muscle spasms	1522	59.3%
Lockjaw	1067	41.6%
Convulsive seizure	1065	41.5%
Difficulty breathing	610	23.8%
Opisthotonos	610	23.8%
Fever	414	16.1%
**Clinical forms of tetanus**		
Generalized	1616	63%
Localized	527	20.5%
Cephalic	188	7.3%
Unknown	236	9.2%
**Grading of disease severity (Ablett grade)**		
I	1021	39.8%
II	692	27%
III	497	19.4%
IV	234	9.1%
Unknown	123	4.7%
**Autonomic nervous disorders**		
None	2300	89.6%
Yes	267	10.4%

Trismus was the predominant presenting symptom, occurring in 2,336 (91.0%) patients. This was followed by dysphagia (63.2%) and muscle spasms (59.3%). The majority of patients (63.0%) were diagnosed with the generalized form of tetanus. Other clinical forms included localized (20.5%) and cephalic (7.3%) tetanus; the clinical form was unknown in 9.2% of cases. According to the Ablett grading system for disease severity, Grade I was the most frequent (39.8%), while severe disease (Grade III and IV) accounted for 28.5% of cases. Autonomic nervous system disorders were present in 267 patients (10.4%).

### Management and outcomes

The management and outcomes for the 2,567 hospitalized tetanus patients are summarized in Table 3. Equine TAT was the most common form of passive immunization, administered to the vast majority of patients (77.6%, n = 1,991). In contrast, human tetanus immunoglobulin (HTIG) was used in a smaller subset of cases (14.5%, n = 372). A total of 204 patients (7.9%) received neither form of anti-tetanus immunoglobulin. Tetanus vaccination was administered to only 19 patients, representing a mere 0.7% of the cohort. Adjunctive medical therapy was widely employed, including antibiotics (93.2%) and sedatives (86.2%); muscle relaxants were used in 12.0% of cases.

**Table 3:** Management and outcomes of tetanus patients

Variable Number (n) Percentage (%)			
**Anti-tetanus immunoglobulin treatment**			
HTIG	372		14.5%
Equine TAT	1991		77.6%
No	204		7.9%
**Tetanus Vaccination**			
Yes	19		0.7%
No	2548		99.3%
**Other treatment medicines**			
Antibiotics	2393		93.2%
Sedative	2214		86.2%
Muscle relaxants	308		12%
**Tracheotomy**			
Yes	393		15.3%
None	2174		84.7%
**Mechanical ventilation**			
Yes	611		23.8%
None	1956		76.2%
**Admission to the ICU**			
Yes	645		25.1%
None	1922		74.9%
**Complication**			
Respiratory complications (pneumonia, respiratory failure)	593		62.2
Cardiovascular complications (tachycardia, hypertension)	40		4.2
Gastrointestinal complications (paralytic ileus)	21		2.2
Renal complications (renal failure)	10		1
Neurological complications (epilepsy)	13		1.4
Other complications	205		21.5
MODS	72		7.5
**Length of hospital stay, in days (median, range)**	12 (0–124)		
**Hospitalization expenses, in RMB (median, range)**	16,110 (7012–34642)		
**Outcome of Admission**			
Recovered	672		26.2%
Improved	1114		43.4%
Discharged without improvement	656		25.6%
Death	125		4.9%

HTIG = Human Tetanus Immunoglobulin

TAT = Tetanus Antitoxin

ICU = Intensive Care Unit

MODS = Multiple Organ Dysfunction Syndrome.

RMB = Renminbi.

Critical care interventions were frequently required. Tracheotomy was performed in 15.3% of patients, and 23.8% required mechanical ventilation. A quarter of all patients (25.1%) were admitted to the Intensive Care Unit (ICU).

Complications were documented in a subset of patients, with respiratory complications (such as pneumonia and respiratory failure) being the most prevalent, accounting for 62.2% of all reported complications. Cardiovascular complications were reported in 4.2% of cases, while multiple organ dysfunction syndrome (MODS) occurred in 7.5%. Other complications involved the gastrointestinal (2.2%), renal (1.0%), and neurological (1.4%) systems.

The median length of hospital stay was 12 days (range: 0–124 days), with a median hospitalization cost of 2,500 RMB (range: 31.35–628,814 RMB). Regarding patient outcomes, the majority were discharged in an improved (43.4%) or recovered (26.2%) condition. However, a significant proportion (25.6%) were discharged without improvement, and the case fatality rate (CFR) was 4.9%.

### Risk factors associated with fatality

Multivariable logistic regression analysis was performed to identify independent risk factors associated with in-hospital fatality among patients with tetanus (Table 4). As anticipated, disease severity was a strong independent predictor of mortality, with significantly increased odds of death observed in patients with Ablett grade II (OR 2.70, 95% CI 1.18–6.17, p = 0.018), grade III (OR 13.40, 95% CI 4.50–39.84, p < 0.001), grade IV (OR 42.73, 95% CI 13.86–131.70, p < 0.001) compared to grade I. Furthermore, the etiology of tetanus was significantly associated with fatality. Notably, injection drug use (OR 5.52, 95% CI 1.11–27.55, p = 0.037) and unknown etiology (OR 1.96, 95% CI 1.14–3.39, p = 0.015) were identified as independent risk factors for death compared to a post-traumatic etiology. Additionally, the presence of comorbidities (OR 1.54, 95% CI 1.03–2.29, p = 0.034) was also independent predictors of mortality. Conversely, admission to the ICU was associated with reduced odds of death (OR 0.47, 95% CI 0.26–0.86, p = 0.015). Case fatality rates stratified by each variable are also presented in Supplementary Table 1.

**Table 4:** Univariate and multivariable analysis of factors associated with case fatality

Variables	Univariate analysis		Multivariable analysis	
	*OR* (*95% CI*)	*P* value^a^	*OR* (*95% CI*)	*P* value
**Gender**				
Female	Ref.^b^		Ref	
Male	1.11 (0.83–1.49)	0.477	1.05 (0.75–1.47)	0.774
**Age**	1.00 (0.99–1.01)	0.781	1.00 (0.98–1.01)	0.437
**Tetanus Vaccination history**				
Yes	Ref		Ref	
No	1.65 (0.43–6.29)	0.46	1.30 (0.37–4.62)	0.683
Unknown	1.23 (0.39–3.87)	0.724	0.86 (0.29–2.54)	0.781
**Comorbidities**				
No	Ref		Ref	
Yes	1.87 (1.32–2.64)	< 0.001	1.54 (1.03–2.29)	0.034
**Etiology of Tetanus**				
Post-traumatic	Ref		Ref	
Non-traumatic infection	1.17 (0.78–1.75)	0.449	1.25 (0.84–1.87)	0.278
Injection drug use	12.73 (3.43–47.21)	< 0.001	5.52 (1.11–27.55)	0.037
Unknown etiology	1.82 (1.22–2.72)	0.003	1.96 (1.14–3.39)	0.015
**Site of Injury or Infection**				
Head and face	Ref		Ref	
Trunk	3.28 (1.25–8.63)	0.016	1.79 (0.56–5.70)	0.327
Upper extremities	1.18 (0.57–2.44)	0.652	1.14 (0.51–2.54)	0.745
Lower extremities	1.01 (0.56–1.83)	0.979	0.85 (0.43–1.70)	0.65
Perineal area	—	/	—	/
Other	2.89 (1.23–6.76)	0.015	1.25 (0.48–3.23)	0.652
Unknown	1.17 (0.57–2.39)	0.664	0.74 (0.31–1.77)	0.5
**Interval from trauma to onset**	1.00 (1.00–1.00)	0.910	1.00 (1.00–1.00)	0.999
**Interval from onset to initial visit**	0.87 (0.79–0.96)	0.005	1.07 (0.60–1.91)	0.818
**Complications**				
No	Ref		Ref	
Yes	1.17 (0.57–2.39)	0.664	1.11 (0.62–1.97)	0.73
**Clinical forms of tetanus**				
Generalized	Ref		Ref	
Localized	0.23 (0.10–0.49)	< 0.001	1.29 (0.55–3.02)	0.564
Cephalic	0.16 (0.05–0.58)	0.005	0.75 (0.18–3.14)	0.692
Unknown	1.17 (0.57–2.41)	0.666	3.28 (1.05–10.19)	0.04
**Autonomic nervous disorders**				
No	Ref		Ref	
Yes	4.14 (2.84–6.04)	< 0.001	1.21 (0.77–1.88)	0.407
**Grading of disease severity (Ablett)**				
I	Ref		Ref	
II	2.78 (1.42–5.44)	0.003	2.70 (1.18–6.17)	0.018
III	12.39 (5.89–26.06)	< 0.001	13.40 (4.50–39.84)	< 0.001
IV	43.38 (20.41–92.22)	< 0.001	42.73 (13.86–131.70)	< 0.001
Unknown	9.88 (3.48–27.99)	< 0.001	3.50 (0.80–15.34)	0.097
**Admission to the ICU**				
No	Ref		Ref	
Yes	1.98 (1.41–2.78)	< 0.001	0.47 (0.26–0.86)	0.015
**Anti-tetanus immunoglobulin treatment**				
HTIG	Ref		Ref	
Equine TAT	1.03 (0.54–1.94)	0.934	1.30 (0.62–2.73)	0.488
HTIG and TAT	0.25 (0.07–0.87)	0.029	0.35 (0.10–1.29)	0.115
No	1.14 (0.52–2.48)	0.742	1.68 (0.64–4.43)	0.292
**Mechanical ventilation**				
No	Ref		Ref	
Yes	3.81 (2.59–5.61)	< 0.001	1.73 (0.95–3.14)	0.075

a. Wald test; Ref. = reference

### Trends in case fatality and hospitalization expenses

**Fig. 2:**
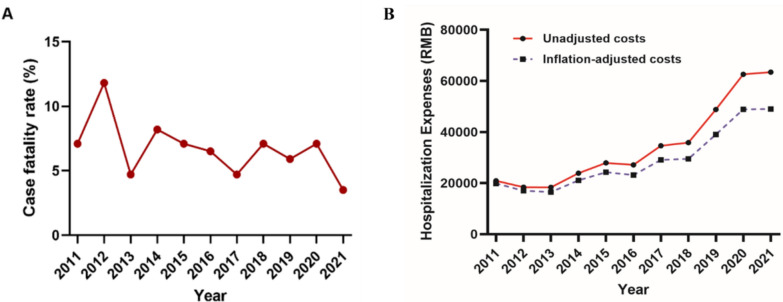
Trends in case fatality and hospitalization expenses (2011–2021). (**A**) The annual case fatality rate among hospitalized tetanus patients (**B**) The median hospitalization expenses (in RMB); Solid line: actual costs unadjusted for inflation. Dashed line: inflation-adjusted costs (with 2010 as the base year, CPI = 100)

An analysis of trends from 2011 to 2021 revealed dynamic changes in both patient outcomes and economic burden. The case fatality rate among hospitalized tetanus patients exhibited a significant declining trend over the decade. The fatality rate peaked at 11.8% in 2012 and fell to its lowest point of 3.5% in 2021, representing a substantial improvement in patient survival. (Fig. 2).

In contrast, the median hospitalization expenses demonstrated a pronounced upward trend during the same period. Costs increased from 20,904 RMB in 2011 to 63,385 RMB in 2021. The trend was consistent after adjusting for inflation using the Consumer Price Index (CPI). This highlights a growing economic burden associated with tetanus hospitalization despite improving clinical outcomes. (Fig. 2).

## Discussion

This study, the largest multicenter retrospective analysis of non-neonatal tetanus to date, reveals an evolving disease landscape in China over a ten-year period. Our comprehensive analysis, conducted in a rapidly economically developing province, not only details clinical features, outcomes, and risk factors for mortality but also identifies three key trends: a persistent hospitalization burden, markedly improved clinical management, and rising medical expenditure.

The study cohort, with a median age of 53 years and predominantly composed of farmers without documented vaccination history, reflects a susceptible generation that grew up before or during the early stages of China’s National Immunization Program, launched in 1978 [13]. This profile signifies a substantial adult population with a historical immunity gap. Against this background, the observed stability in tetanus hospitalization numbers and the increasing proportion of severe cases from 2011 to 2021 should be interpreted. Rather than indicating a failure of the current childhood immunization system, the persistent case burden can be understood as the clinical manifestation of this specific unprotected cohort [14, 15].

This pattern was likely amplified by significant improvements in healthcare access during the study period—a period marked by rapid economic development and healthcare system strengthening in Guangdong Province. As illustrated in Supplementary Fig. 2, substantial growth in GDP, health expenditure per capita, hospital beds, and ICU beds occurred over the decade. Guangdong’s spending on medicine and health increasing at an average annual rate of 17.01%, from 25.3 billion yuan in 2009 to 195.2 billion yuan in 2021 [16]. Furthermore, as the first province in China to integrate basic health insurance for both urban and rural residents in 2012, Guangdong has maintained a participation rate above 95% since 2019 [16, 17]. These developments collectively improved access to tertiary care, effectively "unmasking" the true burden of adult tetanus (particularly its severe forms) by capturing cases that would previously have been managed outside the formal hospital system or gone untreated.

The most encouraging finding of this study is the significant decline in case fatality rates over the decade. This achievement is likely attributable to advancements in specialized medical care, particularly in the domain of intensive care. Our multivariable analysis provides evidence for this: admission to the ICU was identified as an independent protective factor, associated with a 53% reduction in the odds of in-hospital death (OR 0.47). This finding not only demonstrates the effectiveness of local critical care investments but also aligns with the well-established global narrative that ICU availability is a critical determinant of survival, as evidenced by the stark mortality disparities between high-income countries with advanced ICU care and low-income regions without such resources [18].

Our risk factor analysis uncovered that, independent of disease severity, the presumed etiology of tetanus itself is one of the key determinants of outcome. Specifically, injection drug use (IDU) and an unknown etiology were associated with mortality in our multivariable model (OR 5.52 and 1.96, respectively). Several previous reports have focused on the risk of tetanus and its clinical management among injection drug use patients [19, 20]. The risk associated with IDU-related tetanus likely stems from cryptic, contaminated injection sites leading to diagnostic delays, compounded by potential comorbidities and complex social factors. However, as only 11 cases of tetanus associated with IDU were included in our cohort, this finding should be interpreted with caution. Rather than a definitive finding, we consider it an important hypothesis-generating signal. This signal suggests that people who inject drugs may represent an high-risk subgroup in tetanus infection. This mandates a high index of suspicion; clinicians should proactively inquire about drug use in any patient presenting with suggestive symptoms, even in the absence of a clear traumatic injury [19, 21].

Similarly, an unknown etiology should serve as a clinical red flag. It suggests a non-traditional or overlooked portal of entry (e.g., chronic ulcers, otitis media, gastrointestinal foci) that may evade standard diagnostic workup, leading to delays in both diagnosis and crucial wound management. The association with higher mortality underscores the need for exhaustive investigation in every case and implies that the absence of a clear wound should heighten, not lessen, clinical concern.

This study has several limitations that should be considered. First, its retrospective design is inherently susceptible to biases in data recording and the unavailability of certain details. However, the very large sample size and the comprehensive, multicenter nature of our data collection enhance the robustness and generalizability of our principal findings. Second, as our study was restricted to hospitalized patients, those who died before reaching the hospital were excluded. This may lead to an underestimation of the true population-based case fatality rate. Third, the study included only patients aged over 14 years. This focus is justified as the incidence of tetanus in younger cohorts is expected to be very low due to the high coverage of DTP3 vaccination in China since the 1990s.

## Conclusions

This large-scale study reveals a substantial burden of severe non-neonatal tetanus among under-vaccinated adults, persisting despite the region’s economic transition. Case fatality decreased over the study period, likely reflecting improvements in both the accessibility and quality of critical care, albeit with increasing healthcare costs. The association between injection drug use or unknown etiology and mortality warrants heightened clinical vigilance.

## Supplementary Information

Additional file 1.

Additional file 2.

Additional file 3.

## Data Availability

Data from this study are available from the corresponding author upon request. The data are not publicly available because they contain information that could compromise the privacy of the participants.
