# Supplementary figures and images for: 10 years trends and hospitalization outcomes of non-neonatal tetanus: a large-scale multicenter retrospective study in China
Source: Crit Care. 2026 Mar 19;30:320. doi: 10.1186/s13054-026-05931-z (PMC13285191; doi:10.1186/s13054-026-05931-z)

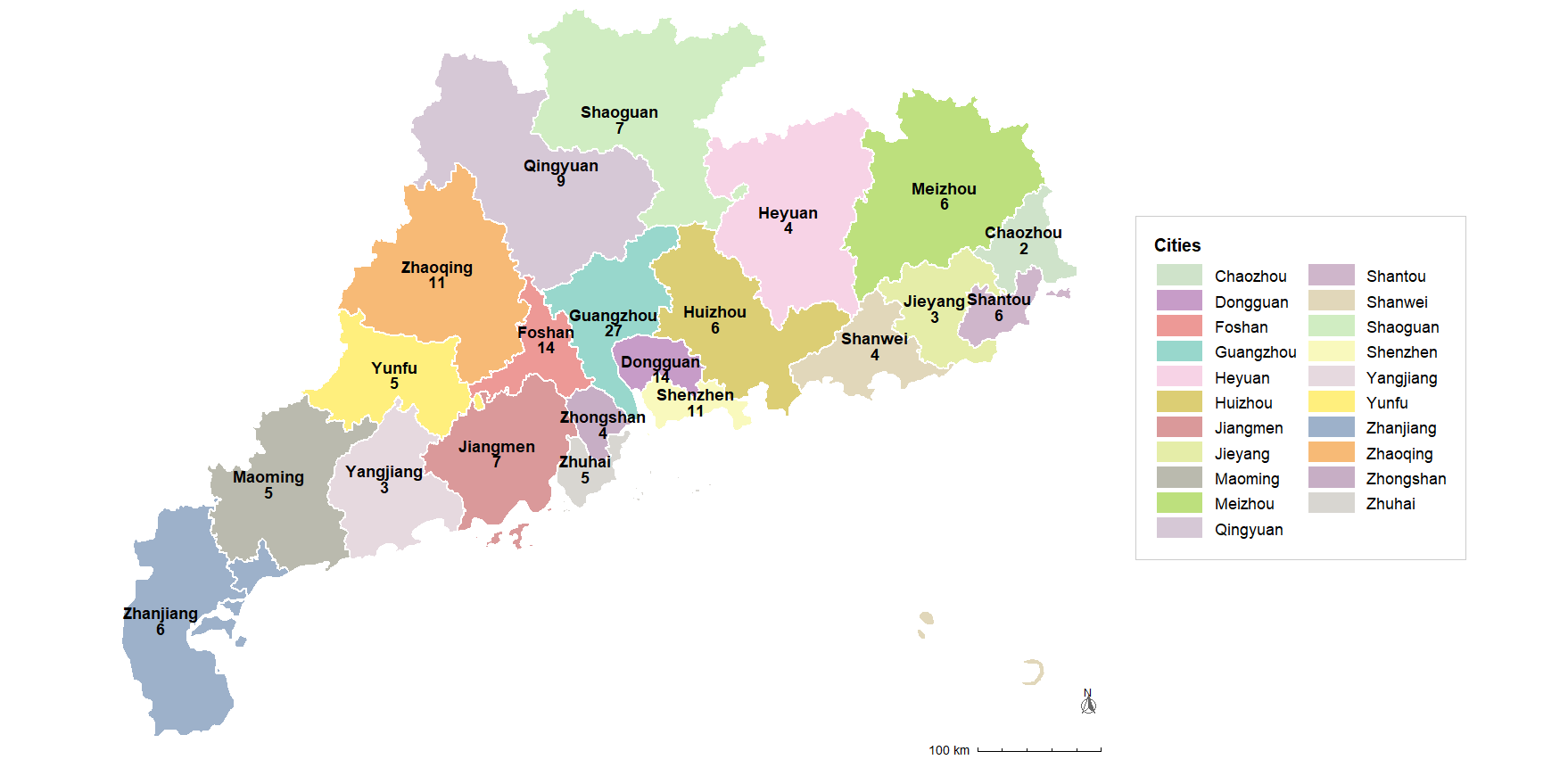

Supplement: Supplementary file 1 — Additional file 1. [file 13054_2026_5931_MOESM1_ESM.tif]

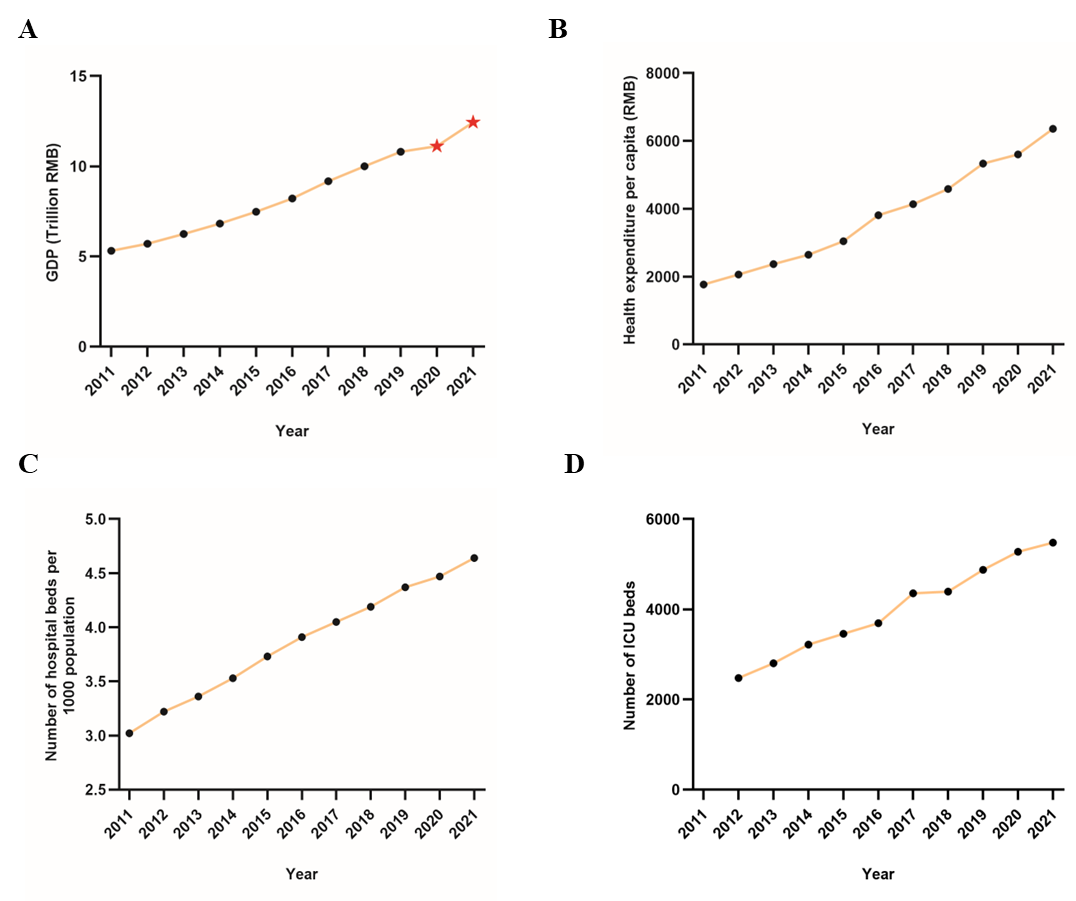

Supplement: Supplementary file 2 — Additional file 2. [file 13054_2026_5931_MOESM2_ESM.tif]
