# Supplementary material for: 10 years trends and hospitalization outcomes of non-neonatal tetanus: a large-scale multicenter retrospective study in China
Source: Crit Care. 2026 Mar 19;30:320. doi: 10.1186/s13054-026-05931-z (PMC13285191; doi:10.1186/s13054-026-05931-z)

(1) **Supplementary Table 1.** Case Fatality Rate Stratified by Each Variable

Variable	Case (n)	Case fatality rate (%)
**Gender**		
Female	45	4.62%
Male	82	5.14%
**Age**		
14~17	1	2.56%
18~45	34	6.31%
46~65	47	4.11%
66~79	32	5.71%
>79	13	4.55%
**Occupation**		
Student	0	0.00%
Labor workers	15	4.40%
Farmer	51	4.54%
Other professions	15	3.11%
The unemployed	39	7.56%
Retiree	7	9.09%
**Tetanus Vaccination history**		
Yes	2	3.64%
None	21	7.05%
Unknown	104	4.70%
**Comorbidities**		
None	64	3.79%
Malignancy	4	9.30%
Hypertension or other cardiovascular disease	17	5.48%
Pulmonary disease	7	11.67%
Diabetes mellitus or other endocrine disease	5	5.21%
Stroke or other neurologic disease	8	10.26%
Renal disease	0	0.00%
Liver and Biliary Diseases	5	7.25%
HIV and other immunodeficiency disorders	5	62.50%
Others	12	5.91%
**Etiology of Tetanus**		
Post-traumatic	43	3.86%
Non-traumatic infection	42	4.61%
Injection drug use	4	36.36%

Variable	Case (n)	Case fatality rate (%)
Unknown etiology	38	7.18%
**Site of Injury or Infection**		
Head and face	8	4.12%
Trunk	7	13.46%
Upper extremities	28	5.02%
Lower extremities	53	4.24%
Perineal area	0	0.00%
Others	10	12.50%
Unknown	21	4.86%
**Presenting symptom**		
Trismus	119	5.09%
Dysphagia	88	5.43%
Muscle spasms	97	6.37%
Lockjaw	87	8.15%
Convulsive seizure	83	7.79%
Difficulty breathing	67	10.98%
Opisthotonos	52	8.52%
Fever	43	10.39%
**Clinical forms of tetanus**		
Generalized	99	6.13%
Localized	8	1.52%
Cephalic	2	1.06%
Unknown	18	7.63%
**Grading of disease severity (Ablett grade)**		
I	6	0.59%
II	13	1.88%
III	40	8.05%
IV	58	24.79%
Unknown	10	8.13%
**Autonomic nervous disorders**		
None	88	3.83%
Yes	39	14.61%
**Anti-tetanus immunoglobulin treatment**		
HTIG	13	3.49%
Equine TAT	94	4.72%
No	127	62.25%
**Tetanus Vaccination**		
Yes	0	0.00%
No	127	4.98%
**Other treatment medicines**		
Antibiotics	118	4.93%
Sedative	112	5.06%
Muscle relaxants	16	5.19%

**Variable**	**Case (n)**	**Case fatality rate (%)**
**Tracheotomy**		
Yes	24	6.11%
None	103	4.74%
**Mechanical ventilation**		
Yes	68	11.13%
None	59	3.02%
**Admission to the ICU**		
Yes	51	7.91%
None	76	3.95%

(2) **Supplementary Figure 1.** Geographic distribution of the 159 participating hospitals across Guangdong Province, China**.**

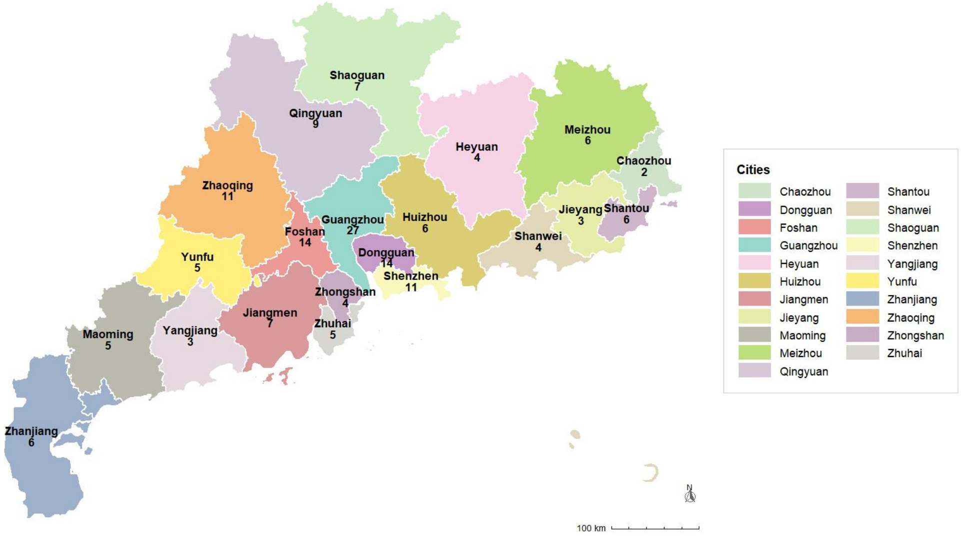

(3) **Supplementary Figure 2.** Evolution of GDP, Health Expenditure, Hospital Beds and ICU Beds in Guangdong Province, China (2011-2021). (A) Gross Domestic Product (GDP) in trillion RMB; Asterisks indicate that high-income status was achieved in 2020 and 2021. (B) Health expenditure per capita (RMB) (C) Number of hospital beds per 1000 population. (D) Number of ICU beds, data only available from 2012 to 2021. Data source: Guangdong Statistical Yearbook and Guangdong Health Statistical Yearbook.

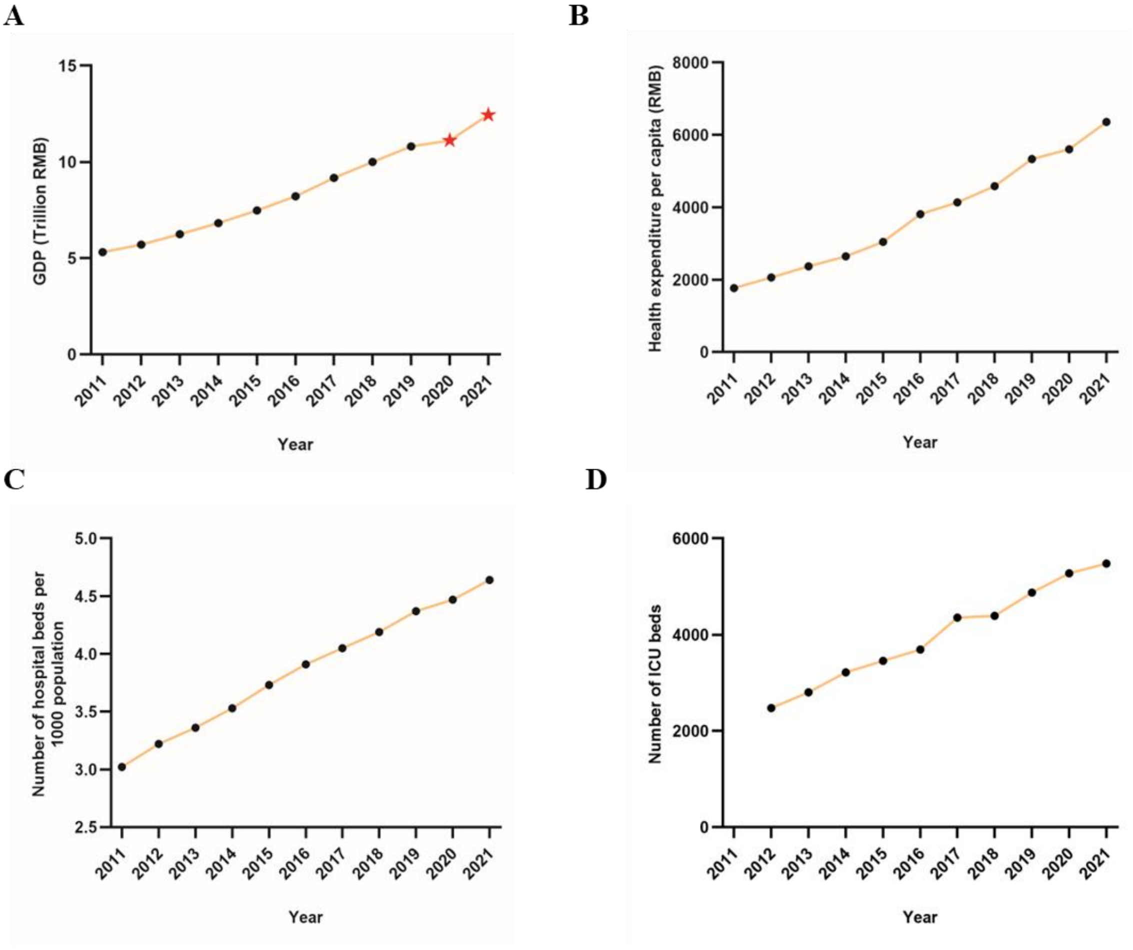

Supplement: Supplementary file 3 — Additional file 3. [file 13054_2026_5931_MOESM3_ESM.pdf]
